# The Pathology of Malaria

**Published:** 1882-03

**Authors:** 


### The Pathology of Malaria.

In the blood of patients suffering from malarial poisoning, M. A. Laveran has found parasitic organisms, very definite in form and most remarkable in character. Some were cylindrical curved bodies, pointed at the extremities, with a delicate outline and a transparent body, colorless except for a blackish spot in the middle, due to pigment granules; on the concave side a fine line could often be traced, which seemed to unite the extremities of the crescent. These bodies presented no movement. Spherical organisms were also seen, transparent, of about the diameter of a red blood-corpuscle, containing pigment grains which, in a state of rest, were often arranged in a definite circle, but sometimes presented rapid movements, and then lost their regular arrangement. On the borders of the spherules very fine filaments could often be perceived in rapid movement. These filaments were in length three or four times the diameter of a red corpuscle. Their number varied. Sometimes three or four were seen around a spherule, to which they communicated an oscillatory movement, displacing the adjacent red corpuscles. The free extremities of the filaments were slightly reflexed. When at rest, the filaments were invisible on account of their tenuity and perfect transparence. These mobile filaments appeared finally by becoming detached from the pigmented spherules, continuing, however, to move freely amidst the corpuscles. There were also bodies of spherical or irregular form, transparent or finely granular, about the hundredth of a micro-millimetre in diameter, containing dark-red, rounded pigment grains, either regularly arranged at the periphery, or aggregated at some part of the spherule. The bodies and granules were both motionless. These appear to be the ultimate or "cadaveric" stage of those last described. They have no nuclei, and do not tint with carmine, a distinction from the pigmented leucocytes, with which they have hitherto been confounded. Lastly, spherical elements were met with similar to those already described, but much smaller in size, and apparently representing a stage in their development. The animated nature of the mobile pigmented spherule, furnished with filaments, appears indisputable. M. Laveran regards it as a form of animalcule, which exists at first in an encysted state, and in the perfect condition becomes free in the form of mobile filaments, a mode of development not uncommon among the lower organisms. Besides these organisms, the blood of patients suffering from malarial fever contain—(1) red corpuscles, which appear to be vacuolated at one or two spots, and contain pigment granules; (2) pigmented leucocytes; (3) free pigment granules, possibly proceeding from the destruction of the parasitical organisms.

These elements were first discovered by M. Laveran a year ago, and since then he has examined the blood in 192 patients affected with various symptoms of malarial poisoning, intermittent and continued fever, and palustral cachexia, and found the organisms in 180. The disease had been contracted for the most part in different regions of Algeria and Tunis. He convinced himself, by numerous and repeated observations, that these organisms are not to be found in the blood of persons suffering from diseases that are not of malarial origin. In most of the cases of malaria in which the examination yielded a negative result, the patient had undergone a course of treatment with quinine, and to this fact the absence of the organisms from the blood was probably due. The addition of a minute quantity of a dilute solution of sulphate of quinine to a drop of blood was found at once to destroy the organisms. In all the examinations great care was taken to preclude the entrance of any extraneous objects into the drop of blood examined. In general the parasitic bodies were found in the blood only at certain times; a little before, and at the moment of the accession of the fever. In some very obstinate cases the organisms were always present in the blood. They rapidly disappeared under the influence of a quinine treatment. It is conjectured that in the apyrexial intervals the organisms probably sojourn in internal organs, especially the spleen and the liver. After death from malarial disease, pigment granules are found in great numbers in the blood, and especially in the small vessels of the spleen and liver; and they may be, in the most severe cases, so abundant that not only the spleen and liver, but the marrow of bone, and even the grey substance of the brain, are darkened by their presence. These pigment granules, which may obstruct the capillary vessels, appear to be derived from the parasitic elements, which perish after death, and become then unrecognizable.—*London Lancet.*

